# The relationship between perceived organizational support and insomnia in Chinese nurses: The Serial multiple mediation analysis

**DOI:** 10.3389/fpsyg.2022.1026317

**Published:** 2022-12-16

**Authors:** Mei-Fang Wang, Qing He, Zhuo Liu, Juan Du

**Affiliations:** ^1^Nursing Department, Xi’an Jiaotong University City College, Xi’an, China; ^2^School of Nursing and Rehabilitation, Xi’an Medical University, Xi’an, China; ^3^School of Nursing, The Fourth Military Medical University, Xi’an, China

**Keywords:** perceived organizational support, psychological capital, occupational stress, effort-reward imbalance, overcommitment, insomnia symptoms

## Abstract

**Background:**

Nurses are in high-pressure, high-load, and high-risk environment for a long time, and their insomnia cannot be ignored. Insomnia not only has a negative impact on the physical and mental health of nurses, but also on the efficiency and quality of nursing work.

**Objective:**

The purpose of this study was to investigate the multiple mediating effect of psychological capital, effort-reward ratio, and overcommitment in the relationship between perceived organizational support and insomnia among Chinese nurses.

**Methods:**

A cross-sectional study has been carried out in a tertiary grade A hospital in Shandong Province, China from June to August 2019. The demographic questionnaire, Perceived Organization Support Questionnaire, Psychological Capital Questionnaire, Chinese version Effort-Reward Imbalance, Questionnaire and Athens Insomnia Scale were used for data collection. SPSS PROCESS 3.4 macro program developed by Hayes was used to test the serial multiple mediation. Descriptive analysis, independent-samples *t*-test, one-way analysis of variance, Pearson’s correlation analyses, ordinary least-squares regression, and the bootstrap method were used for data analysis.

**Results:**

658 valid questionnaires were collected (91.4%). Nurses’ perceived organizational support was positively correlated with psychological capital (*r* = 0.455, *p* < 0.001), and was significantly negatively correlated with effort-reward ratio (*r* = −0.318, *p* < 0.001), overcommitment (*r* = −0.328, *p* < 0.001), and insomnia (*r* = −0.358, *p* < 0.001); Psychological capital was negatively correlated with effort-reward ratio (r = −0.275, *p* < 0.001), overcommitment (*r* = −0.339, *p* < 0.001), and insomnia (*r* = −0.402, *p* < 0.001), respectively; effort-reward ratio and overcommitment were significantly positively correlated with insomnia (*r* = 0.379, *p* < 0.001; *r* = 0.466, *p* < 0.001), respectively. In the model of perceived organizational support—psychological capital—effort-reward ratio—insomnia, the overall mediating effect was −0.080 (95%CI: −0.109 ~ −0.058), and the mediating effect of psychological capital was −0.050, accounting for 34.30% of the total effect; the mediating effect of effort-reward ratio was −0.024, accounting for 16.49% of the total effect; the chain mediating effect of psychological capital and effort-reward ratio was −0.007, accounting for 4.49% of the total effect. In the model of perceived organizational support—psychological capital—overcommitment—insomnia, the overall mediating effect was −0.085 (95%CI: −0.109 ~ −0.064), and the mediating effect of psychological capital was −0.042, accounting for 28.64% of the total effect; the mediating effect of overcommitment was −0.029, accounting for 19.81% of the total effect; the chain mediating effect of psychological capital and overcommitment was −0.015, accounting for 10.14% of the total effect.

**Conclusion:**

Perceived organizational support had direct negative influence on insomnia. Psychological capital and effort-reward ratio/overcommitment acted as chained mediating factor could partially relieve insomnia symptoms related to perceived organizational support. It is suggested to improve the level of organizational support and psychological capital of nurses, and reduce the effort-reward imbalance and overcommitment of nurses, so as to effectively decline and deal with nurses’ insomnia.

## Introduction

Nurses frequently work night shifts and experience psychological and physical effects of their job environment, which often lead them to complain about insomnia. This resulted in enormous psychological stress and an increased likelihood of anxiety, depression, and insomnia (Marvaldi et al., 2021). According to different researchers, the prevalence of insomnia in nurses worldwide was reported to be 30 to 65.4%, while the insomnia rate in Chinese nurses was 37 to 63.9% (Serrano-Ripoll et al., 2021). Insomnia affects the cognitive, physical, and emotional functions of people. This ultimately weakens the immune system, influences metabolism, and increases the risk of mental disorders, depression, coronary heart disease, stroke, morbid obesity, etc. (Marvaldi et al., 2021). Furthermore, studies have found that insomnia can incur economic losses due to reduced enthusiasm and work efficiency, along with increased absenteeism, the incidence of industrial accidents, and medical and healthcare expenses (Sarsour et al., 2011). Additionally, insomnia in nurses can weaken their attention and memory, leading to emotional instability and other psychological disorders, affecting their efficiency and quality of work. More importantly, it can cause nursing errors, accidents, and disputes (Marvaldi et al., 2021; Serrano-Ripoll et al., 2021). Therefore, insomnia in nurses is one of the most concerning health problems. Previous studies on insomnia in the medical field focused mainly on the mechanism and functioning of sleep (Zhang et al., 2020). Some studies were conducted on the factors that influence sleep quality, focusing mainly on the individual characteristics of the respondents, including their general demographic, personality, and work characteristics (Baglioni et al., 2020). However, recent studies on the factors influencing nurses’ insomnia have highlighted the importance of organizational levels, indicating that organizational support is an important factor that affects the quality of nurses’ sleep (Zou et al., 2021).

Perceived organizational support, which considered the general perceptions and beliefs of employees about how the organization viewed their contributions and interests (Eisenberger et al., 2011). It included two core points: One was the employee’s perception of whether the organization valued their contributions, while the other was whether the organization paid attention to the employee’s sense of happiness. Studies have also shown that organizational support reduced psychological and psychiatric responses to stress by representing “the effectiveness of material and emotional support” to meet higher demands at work (Eisenberger et al., 2011; Yoon and Cho, 2022). The hospitals, being the organizational structure of the nursing profession, play an important role in supporting nurses, greatly influencing the whole support system. Therefore, perceived organizational support affects both organizational behavior and the mental health of nurses. Although most studies on organizational support have focused on nurses’ organizational behavior, few other studies have also focused on the impact of perceived organizational support on nurses’ mental health (Abou Hashish, 2017). Fortunately, some existing studies have shown positive relationships between perceived organizational support and nurses’ psychological capital and self-efficacy, along with a negative correlation with nurses’ work pressure, and a tendency toward depression and insomnia (Wang X. et al., 2017).

Luthans developed the concept of psychological capital related to positive organizational behavior and the field of positive psychology (Luthans et al., 2004). Psychological capital is an important positive personal resource that involves self-efficacy, hope, optimism, and resilience (Seligman and Csikszentmihalyi, 2014). Specifically, individuals with high psychological capital possess additional resources to handle their work tasks. They expect good things to happen, quickly recover from setbacks, and are more optimistic about negative situations (Luthans et al., 2004). The psychological state of nursing staff is also an important factor that affects their quality of clinical nursing. A good psychological state plays a positive role in the work of nurses, reduces the occurrence of errors and accidents, and improves the efficiency and quality of nursing work. Additionally, it also facilitates the establishment of a good relationship between nurse–patient and medical care. Furthermore, several models have been proposed. Among them, one is the main effect model, where the psychological capital of individuals, groups, and organizations shows a direct gain function on its outcome variables (Huang et al., 2016). Other buffering and moderate-effect models suggest psychological capital influences outcome variables indirectly. For example, psychological capital has been reported to play a mediating role in relationships between perceived organizational support and job performance, job behavior, job engagement, psychological distress, job burnout, depression, and mental health in nurses (Wang X. et al., 2017; Zhong et al., 2018). Therefore, our first hypothesis proposed that psychological capital may mediate the relationship between perceived organizational support and insomnia.

Occupational stress is measured using the effort-reward imbalance (ERI) model, which focuses on the vital interests of people at work (Wang X. et al., 2017; Cho and Chen, 2020). According to this model, occupational stress occurs when employees perceive an imbalance between their effort and rewards. High efforts and low rewards in the ERI model were reported to significantly predict negative outcomes, including psychological distress, physical symptoms, and low job satisfaction among employees (Huang et al., 2016; Wang X. et al., 2017). The ERI model confirmed the effects of occupational stress on insomnia symptoms among nurses (Huang et al., 2016; Cho and Chen, 2020). Wang et al. showed a correlation between a low level of occupational stress and positive psychological health. Interestingly, several studies have confirmed that perceived organizational support is a predictor of low occupational stress (Wang X. et al., 2017; Hege et al., 2019). Therefore, our second hypothesis proposed that occupational stress may be the potential mediating variable between perceived organizational support and insomnia.

Perceived organizational support was shown to have a positive impact on psychological capital (Wang X. et al., 2017) but a negative association with occupational stress (Wang X. et al., 2017; Yoon and Cho, 2022) and insomnia (Zou et al., 2021). Similarly, psychological capital had a negative correlation with occupational stress (Huang et al., 2016; Wang X. et al., 2017) and insomnia (Huang et al., 2016), while occupational stress was shown to have a positive correlation with insomnia (Huang et al., 2016; Cho and Chen, 2020). Similarly, Zhong et al. indicated that perceived organizational support affected mental health, including psychological distress, through the mediation of psychological capital (Zhong et al., 2018). These findings led to our next hypothesis, which predicted that perceived organizational support was sequentially associated first with increased psychological capital and then with decreased occupational stress, which, in turn, was related to a reduction in insomnia.

Importantly, increased insomnia among nurses not only damaged their physical and mental health but also reduced their efficiency at work and service quality, negatively impacting patient care. Although the effects of perceived organizational support have been explored on insomnia symptoms, only a few studies have emphasized the underlying potential mechanism of the relationship between perceived organizational support and insomnia. Additionally, to fully understand these mechanisms, it is crucial to determine the roles of psychological capital and occupational stress in insomnia. Therefore, we proposed that perceived organizational support not only directly affected insomnia among nurses but also indirectly affected its symptoms. The purpose of this study was to explore the role of psychological capital and occupational stress in the relationship between perceived organizational support and insomnia among Chinese nurses, which included the following four proposed hypotheses: (1) Perceived organizational support would be negatively correlated with insomnia. (2) Psychological capital would play a mediating role in perceived organizational support and insomnia. (3) Occupational stress would play a mediating role between perceived organizational support and insomnia. (4) Psychological capital and occupational stress would act as chain mediators between perceived organizational support and insomnia.

## Materials and methods

### Design and sample

This study was designed as a cross-sectional survey conducted at a tertiary grade-A hospital in Jinan, Shandong Province, China, between June to August 2019. Inclusion criteria for the participants were as follows: (1) Being a registered clinical nurse with work experience of more than 1 year. (2) Informed consent and voluntary participation by the nurse. The exclusion criteria included the following: (1) Nurses being on sick leave or maternity leave during the study period. (2) Being advanced students or on-the-job logistics personnel. Upon the approval of the Institutional Review Board (IRB), uniformly trained investigators sent out anonymous questionnaires to nurses who met the inclusion criteria. Also, standardized explanations were made if necessary. The printed questionnaire was distributed to 720 nurses from which 691 questionnaires were completed and returned (96.0% response rate). The data from 658 participants (91.4% of 720) were analyzed in this study after some questionnaires with missing information were removed.

### Measures

Data collection was carried out using a self-developed demographic questionnaire, Perceived Organization Support Questionnaire (POS), Psychological Capital Questionnaire (PCQ-24), Chinese version Effort-Reward Imbalance (ERI) Questionnaire, and Athens Insomnia Scale (AIS). The average answering time was 10 min.

#### Basic information collection

The self-developed demographic questionnaire included data on gender, age, position, working hours per week, chronic disease, smoking status, night shifts, and negative life events [“Have you had any of the following negative life events in the past year (Life events: refers to the various social changes that people encounter in their daily life): health deterioration, financial difficulties, death of a loved one, loss of possessions, sad events, family conflicts, had difficulties with a workmate, horrors, situations of discrimination, etc.”?].

#### Perceived organizational support measurement

We used a simplified version of the Perceived Organization Support Questionnaire (POS) developed by Eisenberger (Eisenberger et al., 1986). This scale included nine items, with the questionnaire items in each variable scored using a 7-level Likert scale, where 1 represented “complete disagreement” and 7 represented “complete agreement.” Among them, questions 5 and 7 was reversely scored. The higher the score, the stronger the organizational support. The Cronbach’s alpha coefficient of the scale in this study was 0.889, which indicated good reliability of the study.

#### Psychological capital measurement

The psychological capital of nurses was measured using the Psychological Capital Questionnaire (PCQ-24) developed by Luthans (Luthans et al., 2007), which consisted of 24 items. The questionnaire had four dimensions, including self-efficacy, hope, resilience, and optimism for six items each dimension. Each item was scored on a 5-point Likert scale, with ratings between 1 (strongly disagree) to 5 (strongly agree). The sum of all items was regarded as the psychological capital. The higher the score, the higher the psychological capital level of the subjects. Domestic studies have proven good reliability and validity of the Psychological Capital Questionnaire in the cultural background of China (Wang X. et al., 2017). In this study, Cronbach’s alpha coefficient for the scale was 0.956, indicating good reliability.

#### Occupational stress measurement

The ERI questionnaire with 23 items was applied in this study (Li et al., 2005). It consisted of three sub-scales, namely “extrinsic effort” (6 items), “reward” (11 items), and “overcommitment” (6 items). Responses to the items were scored on a 5-point scale where a value of 1 indicated no stressful experience while a value of 5 indicated a highly stressful experience. According to a predefined algorithm, the degree of mismatch between high cost and low gain was quantified by calculating the ratio between the two scales, “effort” and “reward.” Occupational stress is expressed using the effort-reward ratio (ERR) and overcommitment independently. The Chinese version of the ERI scale has shown good reliability and validity and has been widely used in Chinese occupational groups. In this study, the Cronbach’s alpha coefficients for the extrinsic effort, reward, and overcommitment were 0.874, 0.941, and 0.915 respectively, which indicated good reliability of the scale in this study.

#### Insomnia measurement

Insomnia was measured using the Athens Insomnia Scale (AIS) developed by professor DanSedmark (Soldatos et al., 2000). The AIS consists of eight items, where five items assess difficulties encountered with sleep quantity and quality, including sleep induction, awakenings at night, final awakening, total sleep duration, and sleep quality, while the next three items assess daytime consequences of insomnia, including well-being, the functioning capacity, and sleepiness during the day time. Each item was rated on a scale of 0, 1, 2, and 3, which ranged from none to severe. While the score range for insomnia was 0–24, with a higher score indicating more severe insomnia. In this study, the Cronbach’s alpha coefficient was 0.883, which showed good reliability.

### Statistical analyses

Excel 2013 was used for the double-entry of data, and SPSS 25.0 software was used for statistical analysis. Descriptive analysis was represented as counts (*n*) and percentages (%). The demographic data were compared using the independent-sample T-test and one-way analysis of variance (ANOVA). The Least Significant Difference (LSD) method was used for post-comparison between statistically significant multiple groups. Pearson’s correlation analyses were performed on the four variables (perceived organization support, psychological capital, occupational stress, and insomnia). The mediating effects were tested using model 6 of process 3.4 developed by Hayes (Hayes, 2020), where statistically significant confounding factors in the univariate analysis were considered covariates. A value of *p* < 0.05 was considered statistically significant.

### Ethics statement

This study was approved by the Ethics Committee of Shandong Provincial Qianfoshan Hospital. All participants were clearly informed of the purpose, procedures, risks, and benefits related to the study. Moreover, their participation in the research was completely voluntary.

## Results

### Descriptive statistics and univariate analysis

The final participants included 658 nurses. Among them, 109 (16.6%) were males and 549 (83.4%) were females. Age: 473 (71.9%) aged 30 years old, 132 (20.0%) were 30–40 years old, 53 (8.1%) >40 years old; position: 633 (96.2%) were nurses and 25 (3.8%) were head nurses; weekly working hours: 419 (63.7%) 40 h, 239 (36.3%) >40 h; chronic diseases: 528 reported no (80.2%), 130 (19.8%) reported yes; smoking status: 631 (95.9%) never smoked, 12 (1.8%) having given up and 15 smoked (2.3%); night shift work: 489 (74.3%) reported yes and 169 (25.7%) reported no; negative life events: 455 (69.1%) reported no and 203 (30.9%) reported yes.

This study showed significant differences between insomnia and the nurses’ positions (*t* = 2.322, *p* = 0.021), weekly working hours (*t* = −2.027, *p* = 0.043), chronic diseases (*t* = −2.825, *p* = 0.005), smoking statuses (*F* = 4.312, *p* = 0.014), night shifts (*t* = 3.663, *p* <  0.001), and negative life events (*t* = −5.340, *p* < 0.001).

### Correlation analysis

Table 1 shows the average score for insomnia was 7.70 ± 4.62. The correlation matrix for variables is also presented in Table 1. Insomnia was significantly and positively correlated to ERR (*r* = 0.379, *p* < 0.001), and overcommitment (*r* = 0.466, *p* < 0.001). However, it was significantly and negatively correlated to organizational support (*r* = −0.358, *p* < 0.001), and psychological capital (*r* = −0.402, *p* < 0.001).

**Table 1:** Correlation analysis of perceived organization support, psychological capital, occupational stress, and insomnia.

Variables	Organization support	Occupational stress		Psychological capital	Insomnia
		ERR	Overcommitment		
1. Organization support	1				
2. ERR	−0.318***	1			
3. Overcommitment	−0.328***	0.586***	1		
4. Psychological capital	0.455***	−0.275***	−0.339***	1	
5. Insomnia	−0.358***	0.379***	0.466***	−0.402***	1
Mean	45.43	0.76	16.26	101.59	7.70
Standard deviation	9.83	0.42	5.61	17.02	4.62

ERR means effort-reward ratio.

*** *p*<0.001.

### Mediation analysis

**Figure 1:**
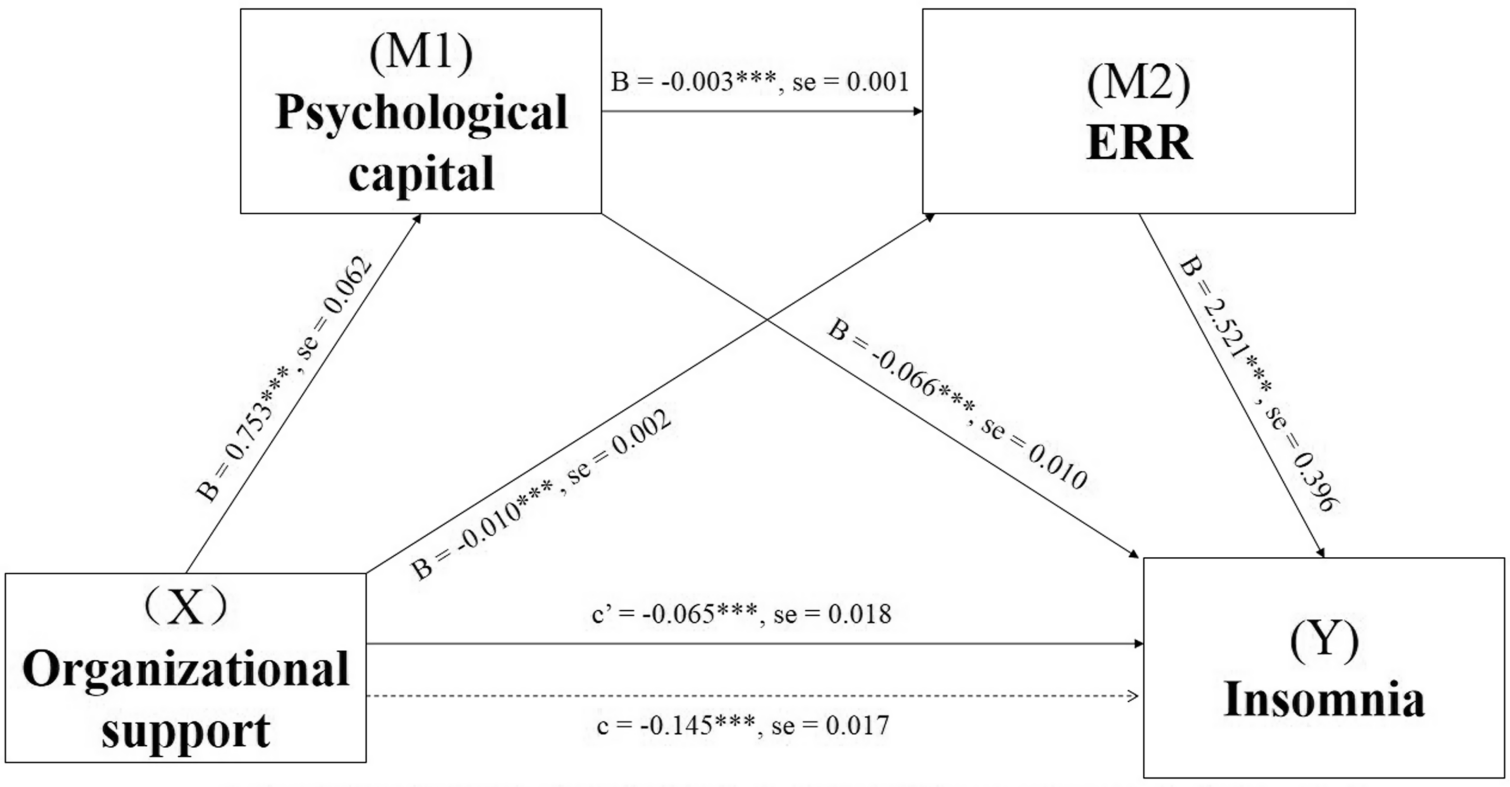
Serial-multiple Mediating effects of psychological capital and ERR between perceived organizational support and insomnia with non-standardized beta values and standard error. ***p < 0.001. ERR means Effort-Reward ratio.

Figure 1 displays the mediating effects of psychological capital and ERR in the relationship between perceived organizational support and insomnia. Both the total effect (*c* = −0.145, SE = 0.017, *p* < 0.001) and direct effect (*c*’ = −0.065, SE = 0.018, *p* < 0.001) of perceived organizational support on insomnia were found to be significant. The direct paths from perceived organizational support to psychological capital (B = 0.753, SE = 0.062, *p* < 0.001) and from perceived organizational support to ERR (B = −0.010, SE = 0.002, *p* < 0.001) were statistically significant. The direct effect of the first mediating variable of the psychological capital was statistically significant on the second mediating variable of ERR (B = −0.003, SE = 0.001, *p* < 0.001). The direct paths from psychological capital to insomnia (B = −0.066, SE = 0.010, *p* < 0.001) and ERR to insomnia (B = 2.521, SE = 0.396, *p* < 0.001) were also statistically significant. The indirect effects obtained by the bootstrap test revealed that the first path was statistically significant from perceived organizational support to insomnia through the first mediator, namely psychological capital (point estimate = −0.050; 95% Boot CI [−0.071, −0.030]). Furthermore, the second path through the second mediator of ERR showed statistical significance (point estimate = −0.024; 95% Boot CI [−0.046, −0.010]). Similarly, the third path through the psychological capital and ERR (point estimate = −0.007; 95% Boot CI [−0.015, −0.001]), as well as the total indirect effect (point estimate = −0.080; 95% Boot CI [−0.109, −0.058]) also showed statistical significance, serially (Table 2).

**Table 2:** Comparison of indirect effects of organizational support on insomnia mediated by psychological capital and ERR.

Effect	Product of coefficients		Bootstrapping 95% BC confidence interval (CI)		Proportion of indirect effect
	Point estimate	Boot SE	BootLL CI	BootUL CI	
Total indirect effect of X on Y	−0.080	0.013	−0.109	−0.058	55.28%
Indirect effect 1: X → M1 → Y	−0.050	0.010	−0.071	−0.030	34.30%
Indirect effect 2: X → M2 → Y	−0.024	0.009	−0.046	−0.010	16.49%
Indirect effect 3: X → M1 → M2 → Y	−0.007	0.004	−0.015	−0.001	4.49%
**Contrasts**					
Model 1 versus Model 2	−0.026	0.016	−0.053	0.008	
Model 1 versus Model 3	−0.043	0.012	−0.066	−0.019	
Model 2 versus Model 3	−0.017	0.008	−0.038	−0.005	

*N* = 658. Number of bootstrap samples for Bias-corrected bootstrap confidence intervals: 50,000. Level of confidence for all confidence intervals: 95%. X = organizational support, M1 = psychological capital, M2 = ERR, Y = insomnia. Model 1 = perceived organizational support–psychological capital–insomnia; Model 2 = perceived organizational support–ERR–insomnia; Model 3 = perceived organizational support–psychological capital–ERR–insomnia. LL = lower level, UL = upper level, ERR means effort-reward ratio.

**Figure 2:**
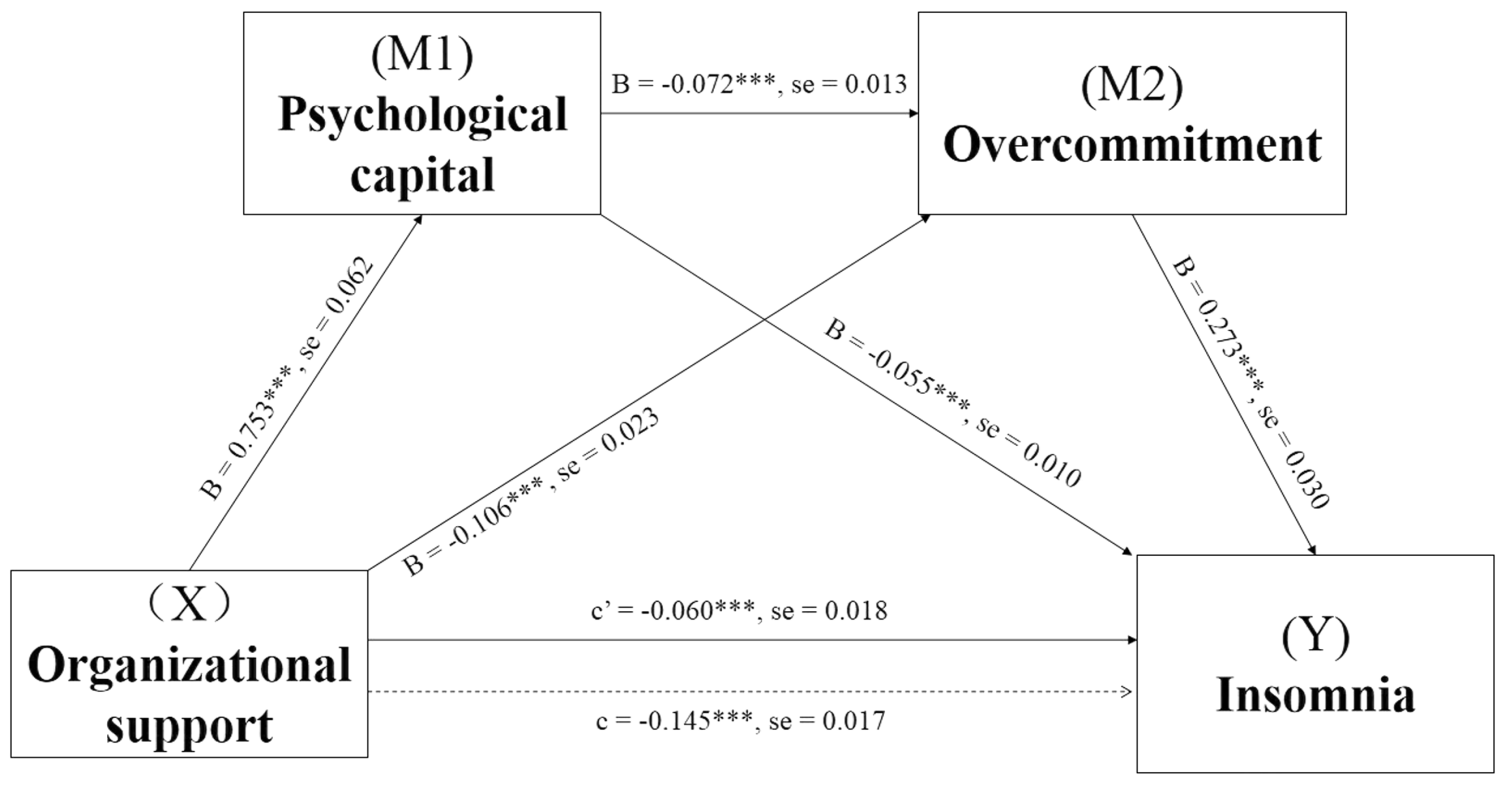
Serial-multiple Mediating effects of psychological capital and overcommitment between perceived organizational support and insomnia with non-standardized beta values and standard error. ***p < 0.001.

Figure 2 displays the mediating effects of psychological capital and overcommitment in the relationship between perceived organizational support and insomnia. Both the total effect (c = −0.145, SE = 0.017, *p* < 0.001) and direct effect (c’ = −0.060, SE = 0.018, *p* < 0.001) of perceived organizational support on insomnia were found to be significant. The direct paths from perceived organizational support to psychological capital (B = 0.753, SE = 0.062, *p* < 0.001) and perceived organizational support to overcommitment (B = −0.106, SE = 0.023, *p* < 0.001) showed statistical significance. The direct effect of psychological capital as the first mediating variable was also significant on the second mediating variable of overcommitment (B = −0.072, SE = 0.013, *p* < 0.001). The direct paths from psychological capital to insomnia (B = −0.055, SE = 0.010, *p* < 0.001) and overcommitment to insomnia (B = 0.273, SE = 0.030, *p* < 0.001) also showed statistical significance. The indirect effects obtained by the bootstrap test showed that the first path from perceived organizational support to insomnia through the first mediator, namely psychological capital (point estimate = −0.042; 95% Boot CI [−0.062, −0.024]), showed statistical significance. Similarly, the second path through the second mediator of overcommitment (point estimate = −0.029; 95% Boot CI [−0.046, −0.014]) was also statistically significant. Moreover, the third path through the psychological capital and overcommitment (point estimate = −0.015; 95% Boot CI [−0.022, −0.008]), as well as the total indirect effect was also statistically significant (point estimate = −0.085; 95% Boot CI [−0.109, −0.064]), serially (Table 3).

**Table 3:** Comparison of indirect effects of organizational support on insomnia mediated by psychological capital and overcommitment.

Effect	Product of coefficients		Bootstrapping 95% BC confidence interval (CI)		Proportion of indirect effect
	Point estimate	Boot SE	BootLL CI	BootUL CI	
Total indirect effect of X on Y	−0.085	0.012	−0.109	−0.064	58.59%
Indirect effect 1: X → M1 → Y	−0.042	0.010	−0.062	−0.024	28.64%
Indirect effect 2: X → M2 → Y	−0.029	0.008	−0.046	−0.014	19.81%
Indirect effect 3: X → M1 → M2 → Y	−0.015	0.004	−0.022	−0.008	10.14%
**Contrasts**					
Model 1 versus Model 2	−0.013	0.013	−0.039	0.013	
Model 1 versus Model 3	−0.027	0.010	−0.048	−0.007	
Model 2 versus Model 3	−0.014	0.009	−0.034	0.003	

*N* = 658. Number of bootstrap samples for Bias-corrected bootstrap confidence intervals: 50,000. Level of confidence for all confidence intervals: 95%. X = organizational support, M1 = psychological capital, M2 = overcommitment, Y = insomnia. Model 1 = perceived organizational support–psychological capital–insomnia; Model 2 = perceived organizational support–overcommitment–insomnia; Model 3 = perceived organizational support–psychological capital–overcommitment–insomnia. LL = lower level, UL = upper level.

Contrasting findings were analyzed in deciding whether specific indirect effects of mediators were stronger than the others. In Table 2 we found that two pairs of contrasts were not inside the zero-point estimate based on the 95% Boot CI, which indicated statistical differences between the two indirect effect pathways. It showed that the path through single mediation by psychological capital or ERR exerted stronger mediating power than the serial-multiple mediations of psychological capital and ERR. Similarly, Table 3 shows another comparison, where the psychological capital still exerted a stronger mediation effect than the serial-multiple mediation of psychological capital and overcommitment.

## Discussion

This study revealed that nurses who worked for more than 40 h weekly, suffered chronic diseases, smoked, worked night shifts, or had negative life events, reported more obvious insomnia symptoms. This was similar to the results of previous research, where the socio-demographic and work-related factors contributed significantly to insomnia in healthcare workers (Zou et al., 2021). Such factors are related to lower sleep quality and should be noted as early alerts.

A strong association was displayed between perceived organizational support and insomnia. The results showed a direct effect, which accounted for nearly half of the association in both models, irrespective of whether the second mediating variable was ERR or overcommitment. We found that increasing organizational support perceived by nurses indicated a decrease in their risk of insomnia. This was supported by the results of the research conducted on healthcare workers during the COVID-19 epidemic (Zou et al., 2021). Organizational support was reported to modify the attitudes and behaviors of workers. A low perceived organizational support resulted in work-related stress and a negative feeling (Kocoglu Sazkaya and Gormezoglu, 2021). A study conducted on a service organization revealed that the failure of a corporation to fulfill obligations of reciprocity acted as a stressor in employees, which increased their psychological distress, leading to insomnia (Garcia et al., 2018).

In this study, psychological capital played a dominant mediating role in the association between perceived organizational support and insomnia. When the second mediating variable was ERR, the mediating effect of psychological capital was 34.30% of the total effect of the perceived organizational support on insomnia, but if the mediating variable was overcommitment, it accounted for 28.64% of the total effect. Our results indicated that the stronger the organizational support was perceived by nurses, the higher was their psychological capital, which led to a reduction in insomnia. Many studies showed that better social support improved sleep quality by developing mental resilience (Lei et al., 2021) and reducing negative emotions (Grey et al., 2020). The role of positive psychology is extended in this study. Throughout personal growth, psychological capital remains a relatively stable positive psychological ability. It provides an important buffer between external situations and internal perception. Specifically, if the level of perceived organizational support was low, the sense of threat and vulnerability from even a single social network affected sleep quality (Evans et al., 2016). However, organizational support provides a safe and relaxed context that, according to transition stress theory, enables individuals to face and overcome difficulties more courageously and confidently. Moreover, a higher psychological capital makes it easier for individuals to recover from negative situations by experiencing more emotional support, which helps them to get rid of their negative state and fall asleep easily (Lv et al., 2022). Optimism, one dimension of the psychological capital, is the most important positive resource for combating these negative emotions (Wang et al., 2021).

We identified two sub-variables of occupational stress mediating the relationship between perceived organizational support and insomnia. One mediating variable was the ERR, which explained 16.49% of the effect between perceived organizational support and insomnia. A higher perceived organizational support reduced the ERR, which, in turn, promoted the nurses’ sleep. Several studies have also demonstrated that organizational support was negatively correlated with the ERR. When the perceived organizational support was high, in-role performance and extra-role performance of employees were improved, naturally balancing the efforts and rewards (Zaman et al., 2019). However, if the perceived organizational support was low, employees realized that organization paid insufficient attention to their contributions and interests, which was manifested as an imbalance between efforts and rewards, increasing the work-related stress (Baek et al., 2021). Moreover, several studies have suggested that high ERR affected the health and productivity of individuals in the workplace (Georg et al., 2019) or school (Hodge et al., 2020), which impaired their sleep quality. The other mediating variable is overcommitment, accounting for 19.81% of the total effect. If the perceived organizational support was higher, the overcommitment of nurses was reduced, resulting in better sleep. A negative correlation between perceived organizational support and overcommitment was an interesting finding in this study. We believe that overcommitment usually refers to a condition of overload, where the challenges of the job are underestimated, but the available resources are overestimated (Violanti et al., 2018). A lower perceived organizational support was more likely to stimulate the overcommitment of employees. Contrastingly, a higher perceived organizational support led the employees to lower their guard and respond more freely to various situations at work. Furthermore, overcommitment was found to be negatively correlated with well-being and to increase burnout and stress, making employees susceptible to insomnia (Hodge et al., 2020). Perceived control over work is a key factor affecting workers’ perception of work stress.

We defined the multiple mediating effects of psychological capital and occupational stress on the relationship between perceived organizational support and insomnia. Positive organizational support was first associated with increased psychological capital and then with decreased ERR or overcommitment, which, in turn, was related to a reduction in insomnia. The chain mediating effect related to ERR or overcommitment accounted for 4.49% and 10.14% of the total effect, respectively. Most previous studies have focused mainly on the role of occupational stress in psychological capital. Psychological capital, a potentially positive resource, has been reported as a mediator between occupational stress and job burnout (Li et al., 2015), fatigue (Tian et al., 2020), or depression (Liu et al., 2012). However, in this study, we reversed the relationship and emphasized that the negative relationship between psychological capital and occupational stress was significant. Some scholars have also put forward the stressor load-stress resilience model (SLSRM), which revealed that positive personal characteristics, including psychological capital, may determine the individual stress resilience, affecting the physical or mental health via the adoption of different coping strategies (Wang L. et al., 2017). Psychological capital is a combination of efficacy, optimism, hope, and resilience. People with high psychological capital can bear stress more readily and maintain physical and psychological well-being and happiness in negative situations (Riolli et al., 2012). Also, if people believe that they lack resources (both psychological and social aspects) to deal with difficult events, they actually experience more stress (Han et al., 2019).

The findings of this study have important implications in practice. Hospital managers in China need full awareness of the prevalence of insomnia among nurses. As front-line clinical workers, the sleep quality of nurses not only affects their own health but also affects the medical safety of patients. Therefore, managers should pay more attention to the sleep situation of nurses. A combination of insufficient organizational support, low personal-psychological capital, strong occupational stress, and insomnia in nurses can be a particularly serious problem. To improve insomnia, managers need to take measures from the following three aspects: organizational support, psychological capital, and occupational stress. Managers need to provide equal opportunities and respond to the efforts of every nurse with instrumental and emotional organizational support. Group counseling and psychological training need to be provided to nurses to promote the formation of positive psychological capital. Regarding ERR, managers should balance the demand and reward of the job by not only reducing bureaucratic workloads, optimizing work content, and arranging shifts reasonably but also by giving full attention to their income, promotion, respect, and other factors. Additionally, while dealing with various tasks, nurses themselves should avoid overcommitment by adopting effective strategies (e.g., time management).

## Conclusion

The present study was the first to explore the relationship between perceived organizational support and insomnia symptoms among Chinese nurses using a serial-multiple mediation model. The results of the present study showed that psychological capital and occupational stress played chain mediating effect between perceived organizational support and insomnia in nurses. Specifically, increased perceived organizational support was sequentially associated with increased psychological capital first and then decreased occupational stress, which was, in turn, related to reduced insomnia symptoms. Therefore, paying attention to welfare, creating supportive working environment and investing to improve psychological capital, decreasing occupational stress can help alleviate insomnia symptoms of nurses.

## Strengths and limitations

Previous studies of the relationship between organizational support and sleep problems have focused more on the direct effects, while studies of its internal mediating mechanisms have been relatively lacking. In this study, nurses were taken as the research object, and the mechanism of perceived organizational support on insomnia was discussed from the personal level (psychological capital) and the organization level (occupational stress), so as to broaden the research on the complex mediating variables of the relationship between perceived organizational support and insomnia. It is expected that clinical nursing managers and nurses can effectively alter the status quo of nurses’ insomnia through the intervention of mediating variables, to promote nurses’ physical and mental health and improve the quality of nursing. There are still some limitations in this study. First, the method employed in this study is a cross-sectional survey, so the conclusions obtained are difficult to infer causally and can only be a description of the current situation or a description of a mutual relationship. Second, although quality is controlled in surveys, self-report bias can occur due to the strong subjectivity of self-filling questionnaires and psychological questionnaires. Third, because 4.8% of the questionnaires had more than 10% missing data or substantial missing general demographic data, we were unable to determine the statistical difference between the censored data and the valid questionnaires. Therefore, the conclusions of this study can only be used as a description of the current situation, and extrapolation is limited to a certain extent. In the future, on the one hand, longitudinal studies will be conducted to verify the causal relationship between these variables and the effects of increased and decreased perceived organizational support, psychological capital, and occupational stress on insomnia. On the other hand, quality control efforts will be intensified to make the questionnaire more complete and effective.

## Data availability statement

The supporting data for this article can be obtained by contacting the corresponding author by email with a sufficient reason.

## Author contributions

M-FW and JD conceived and designed the research. M-FW, JD, and ZL performed the research and contributed to data analyses. M-FW, QH, and JD wrote the paper. All authors contributed to the article and approved the submitted version.

## Funding

This work was supported by 2021 Key scientific Research Project of Xi’an Jiaotong University City College [grant number 2021Z04].

## Conflict of interest

The authors declare that the research was conducted in the absence of any commercial or financial relationships that could be construed as a potential conflict of interest.

## Publisher’s note

All claims expressed in this article are solely those of the authors and do not necessarily represent those of their affiliated organizations, or those of the publisher, the editors and the reviewers. Any product that may be evaluated in this article, or claim that may be made by its manufacturer, is not guaranteed or endorsed by the publisher.
