# Corrigendum: The relationship between perceived organizational support and insomnia in Chinese nurses: the serial multiple mediation analysis

**DOI:** 10.3389/fpsyg.2023.1239608

**Published:** 2024-07-30

**Authors:** Mei-Fang Wang, Qing He, Zhuo Liu, Juan Du

**Affiliations:** ^1^Nursing Department, Xi'an Jiaotong University City College, Xi'an, China; ^2^School of Nursing and Rehabilitation, Xi'an Medical University, Xi'an, China; ^3^School of Nursing, The Fourth Military Medical University, Xi'an, China

**Keywords:** perceived organizational support, psychological capital, occupational stress, effort-reward imbalance, overcommitment, insomnia symptoms

In the published article, there was an error in the author list. Authors Yan-Ling Du, Chao Wu and Hong-Juan Lang were erroneously included in the author list. The corrected author list appears below.

Mei-Fang Wang^1†^, Qing He^2†^, Zhuo Liu^3^ and Juan Du^3*^

The corrected article citation appears below.

Wang M-F, He Q, Liu Z and Du J (2022) The relationship between perceived organizational support and insomnia in Chinese nurses: the serial multiple mediation analysis. *Front. Psychol*. 13:1026317. 10.3389/fpsyg.2022.1026317

The corrected copyright statement is shown below.

© 2022 Wang, He, Liu and Du. This is an open-access article distributed under the terms of the Creative Commons Attribution License (CC BY). The use, distribution or reproduction in other forums is permitted, provided the original author(s) and the copyright owner(s) are credited and that the original publication in this journal is cited, in accordance with accepted academic practice. No use, distribution or reproduction is permitted which does not comply with these terms.

The corrected Author contributions appears below.

**Author contributions**

M-FW and JD conceived and designed the research. M-FW, JD, and ZL performed the research and contributed to data analyses. M-FW, QH, and JD wrote the paper. All authors contributed to the article and approved the submitted version.

Any other relevant parts of the original article have also been updated to reflect this authorship change.

Additionally, in the published article, there were several errors in the text.

Firstly, the questionnaire was issued from June to August 2019 instead of March to May 2021, and 720 questionnaires were issued, not 810.

A correction has been made to *Abstract, Methods*. This sentence previously stated:

“A cross-sectional study has been carried out in a tertiary grade A hospital in Shandong Province, China from March 2021 to May 2021.”

The corrected sentence appears below:

“A cross-sectional study has been carried out in a tertiary grade A hospital in Shandong Province, China from June to August 2019.”

A correction has been made to *Abstract, Results*. This sentence previously stated:

“658 valid questionnaires were collected (81.2%).”

The corrected sentence appears below:

“658 valid questionnaires were collected (91.4%).”

Corrections have been made to *Materials and methods, Design and sample, Paragraph 1*. One sentence previously stated:

“This study was designed as a cross-sectional survey conducted at a tertiary grade-A hospital in Jinan, Shandong Province, China, between March and May 2021.”

The corrected sentence appears below:

“This study was designed as a cross-sectional survey conducted at a tertiary grade-A hospital in Jinan, Shandong Province, China, between June to August 2019.”

Another part of this paragraph previously stated:

“The printed questionnaire was distributed to 810 nurses from which 756 questionnaires were completed and returned (93.3% response rate). Ninety-eight subjects (13.0% of 756) with missing values for demographic information, perceived organizational support, psychological capital, occupational stress, and insomnia items were excluded, of which missing data was greater than 10%, or general demographic data was severely missing. There were also six questionnaires with less than 10% missing values, and the mean values were taken for data analysis. Finally, the data from 658 subjects (81.2% of 810, 87.0% of 756) were analyzed in this study.”

The corrected text appears below:

“The printed questionnaire was distributed to 720 nurses from which 691 questionnaires were completed and returned (96.0% response rate). The data from 658 participants (91.4% of 720) were analyzed in this study after some questionnaires with missing information were removed.”

As such, the whole corrected paragraph should now be written as shown below.

“This study was designed as a cross-sectional survey conducted at a tertiary grade-A hospital in Jinan, Shandong Province, China, between June to August 2019. Inclusion criteria for the participants were as follows: (1) Being a registered clinical nurse with work experience of more than 1 year. (2) Informed consent and voluntary participation by the nurse. The exclusion criteria included the following: (1) Nurses being on sick leave or maternity leave during the study period. (2) Being advanced students or on-the-job logistics personnel. Upon the approval of the Institutional Review Board (IRB), uniformly trained investigators sent out anonymous questionnaires to nurses who met the inclusion criteria. Also, standardized explanations were made if necessary. The printed questionnaire was distributed to 720 nurses from which 691 questionnaires were completed and returned (96.0% response rate). The data from 658 participants (91.4% of 720) were analyzed in this study after some questionnaires with missing information were removed.”

A correction has been made to *Strengths and limitations*. This sentence previously stated:

“Third, because 13.0% of the questionnaires had more than 10% missing data or substantial missing general demographic data, we were unable to determine the statistical difference between the censored data and the valid questionnaires.”

The corrected sentence appears below:

“Third, because 4.8% of the questionnaires had more than 10% missing data or substantial missing general demographic data, we were unable to determine the statistical difference between the censored data and the valid questionnaires.”

There were also small grammatical errors in the *Results* and *Discussion* sections.

Instead of “from the second mediating variable,” it should be “on the second mediating variable.” Corrections have been made to *Results, Mediation analysis, Paragraphs 1* and *2*. One sentence previously stated:

“The direct effect of the first mediating variable of the psychological capital was statistically significant from the second mediating variable of ERR (B = −0.003, SE = 0.001, *p* < 0.001).”

The corrected sentence appears below:

“The direct effect of the first mediating variable of the psychological capital was statistically significant on the second mediating variable of ERR (*B* = −0.003, SE = 0.001, *p* < 0.001).”

Another sentence previously stated:

“The direct effect of psychological capital as the first mediating variable was also significant from the second mediating variable of overcommitment (B = −0.072, SE = 0.013, p < 0.001).”

The corrected sentence appears below:

“The direct effect of psychological capital as the first mediating variable was also significant on the second mediating variable of overcommitment (*B* = −0.072, SE = 0.013, *p* < 0.001).”

A correction has been made to *Discussion, Paragraph 2*. This sentence previously stated:

“This was supported by the results of previous research conducted on healthcare workers during the COVID-19 epidemic (Zou et al., 2021).”

The corrected sentence appears below:

“This was supported by the results of the research conducted on healthcare workers during the COVID-19 epidemic (Zou et al., 2021).”

Additionally, since the data was collected from June to August 2019, when the COVID-19 pandemic did not occur, there are errors in the *Introduction* and *Discussion* where the COVID-19 pandemic is mentioned.

A correction has been made to *Introduction, Paragraph 1*. This sentence previously stated:

“In particular, during the 2019 coronavirus disease pandemic, the nursing job, in many cases, required completion in high-pressure and high-risk environments with severe workloads.”

This sentence should be removed.

A correction has been made to *Discussion, Paragraph 1*. The section of text concerned previously stated:

“During the COVID-19 outbreak, the quality of sleep of nursing staff was significantly lower than that of other healthcare workers (Zare et al., 2022). When healthcare workers returned to work 3 months after SARS-CoV-2 infection, one-third of them reported worsening sleep patterns (Grazzini et al., 2022). It is not just common risk factors such as female sex, advanced age, persistent fatigue, and circadian rhythm changes that can cause insomnia. Epidemic-related factors including disease trauma, personal isolation, family history of COVID-19, and the death of a family member due to COVID-19 further contribute to insomnia among healthcare workers during the COVID-19 epidemic.”

This text should be removed.

A correction has been made to *Discussion, Paragraph 3*. The section of text concerned previously stated:

“During the COVID-19 epidemic, unprecedented population restrictions, history of COVID-19 in family members, death of family members by COVID-19, etc., have brought additional stress, anxiety, and depression to nursing staff during this stage, which will lead to shorter sleep duration and lower sleep quality (Zare et al., 2022). Studies have defined the sleep disorder associated with the COVID-19 pandemic as “COVID-somnia” (Özçelik et al., 2022).”

This text should be removed.

A correction has been made to *Discussion, Paragraph 4*. This sentence previously stated:

“Research suggested that during the COVID-19 pandemic, atypical work schedules such as shift work status and extended working hours may create greater uncertainty for the healthcare workers, which will affect their sleep (Power et al., 2022).”

This sentence should be removed.

A correction has been made to *Discussion, Paragraph 6*. The section of text concerned previously stated:

“It is essential to note that during the COVID-19 pandemic, numerous things can be digitized, such as virtual clinics, psychotherapy or psycho-education delivered remotely, to support the mental health of healthcare workers (Pappa et al., 2020). A mobile app providing breathwork, yogic practices, and meditation sessions has been shown to be effective in improving insomnia among healthcare workers, which may work through a physiological mechanism involving the autonomic nervous system (Currie et al., 2022).”

This text should be removed.

In light of the corrections made to the text, the following references have been removed:

Currie, K., Gupta, B. V., Shivanand, I., Desai, A., Bhatt, S., Tunuguntla, H. S., et al. (2022). Reductions in anxiety, depression and insomnia in health care workers using a non-pharmaceutical intervention. *Front. Psychol*. 13:983165. 10.3389/fpsyt.2022.983165

Grazzini, M., Lulli, L. G., Mucci, N., Paolini, D., Baldassarre, A., Gallinoro, V., et al. (2022). Return to work of healthcare workers after SARS-CoV-2 infection: determinants of physical and mental health. *Int. J. Environ. Res. Public Health* 19:6811. 10.3390/ijerph19116811

Özçelik, N., Kesin, H. V., Telatar, G., Özyurt, S., Yilmaz Kara, B., Gümüş A., et al. (2022). ‘COVID-Somnia' in healthcare workers during the pandemic. *Hosp. Pract*. 50, 273–281. 10.1080/21548331.2022.2102777

Pappa, S., Ntella, V., Giannakas, T., Giannakoulis, V. G., Papoutsi, E., and Katsaounou, P. (2020). Prevalence of depression, anxiety, and insomnia among healthcare workers during the COVID-19 pandemic: a systematic review and meta-analysis. *Brain Behav. Immun*. 88, 901–907. 10.1016/j.bbi.2020.05.026

Power, N., Perreault, M., Ferrari, M., Boudreau, P., and Boivin, D. B. (2022). Sleep of healthcare workers during the COVID-19 pandemic and the role of atypical work schedules: a scoping review. *J. Biol. Rhythm*. 37, 358–384. 10.1177/07487304221103376

Zare, F., Sadeghian, F., Alatab, S., Chaman, R., and Mirrezaie, S. M. (2022). COVID-19 epidemic effects on sleep quality among health sector workers: a follow up study. *Chronobiol. Int*. 39, 1015–1026. 10.1080/07420528.2022.2058402

Additionally, in the published article, there was an error in the Funding statement. The text regarding the “Special Research Project of Health Care of Air Force Military Medical University [grant number 22KYBJ01]” was wrongly included. The correct Funding statement appears below.

**FUNDING**

This work was supported by 2021 Key scientific Research Project of Xi'an Jiaotong University City College [grant number 2021Z04].

Additionally, in the published article, there was an error in Table 1 as published. Some of the general demographic data in Table 1 was duplicated from previously published research and should be removed. The data also appears in “Wang MF, Shao P, Wu C, Zhang LY, Zhang LF, Liang J, Du J. The relationship between occupational stressors and insomnia in hospital nurses: The mediating role of psychological capital. Front Psychol. 2023 Feb 14;13:1070809. 10.3389/fpsyg.2022.1070809”. To rectify this, the general demographic data should be described in the results, and then Table 1 should be removed.

A correction has been made to *Results, Descriptive statistics and univariate analysis, Paragraphs 1 and 2*. These paragraphs previously stated:

“The final participants included 658 nurses, where 83.4% were female, 71.9% were less than 30 years old, 96.2% were nurses in position, 36.3% worked more than 40 h per week, 19.8% suffered from chronic diseases, 95.9% never smoked, 74.3% had night shifts, and 30.9% experienced negative life events (Table 1).

Table 1 shows significant differences between insomnia and the nurses' positions (t = 2.322, p = 0.021), weekly working hours (t = −2.027, p = 0.043), chronic diseases (t = −2.825, p = 0.005), smoking statuses (F = 4.312, p = 0.014), night shifts (t = 3.663, p < 0.001), and negative life events (t = −5.340, p < 0.001).”

The corrected paragraphs appear below.

“The final participants included 658 nurses. Among them, 109 (16.6%) were males and 549 (83.4%) were females. Age: 473 (71.9%) aged ≤ 30 years old, 132 (20.0%) were 30–40 years old, 53 (8.1%) >40 years old; position: 633 (96.2%) were nurses and 25 (3.8%) were head nurses; weekly working hours: 419 (63.7%) ≤ 40 h, 239 (36.3%) >40 h; chronic diseases: 528 reported no (80.2%), 130 (19.8%) reported yes; smoking status: 631 (95.9%) never smoked, 12 (1.8%) having given up and 15 smoked (2.3%); night shift work: 489 (74.3%) reported yes and 169 (25.7%) reported no; negative life events: 455(69.1%) reported no and 203 (30.9%) reported yes.

This study showed significant differences between insomnia and the nurses' positions (*t* = 2.322, *p* = 0.021), weekly working hours (*t* = −2.027, *p* = 0.043), chronic diseases (*t* = −2.825, *p* = 0.005), smoking statuses (*F* = 4.312, *p* = 0.014), night shifts (*t* = 3.663, *p* < 0.001), and negative life events (*t* = −5.340, *p* < 0.001).”

Table 1 should then be removed, and the remaining tables and their corresponding citations should be updated accordingly.

The authors apologize for these errors and state that they do not change the scientific conclusions of the article in any way. The original article has been updated.
